# Purification and Identification of miRNA Target Sites in Genome Using DNA Affinity Precipitation

**DOI:** 10.3389/fgene.2019.00778

**Published:** 2019-09-12

**Authors:** Yu Xun, Yingxin Tang, Linmin Hu, Hui Xiao, Shengwen Long, Mengting Gong, Chenxi Wei, Ke Wei, Shuanglin Xiang

**Affiliations:** ^1^Key Laboratory of Protein Chemistry and Developmental Biology of Education Ministry of China, College of Life Science, Hunan Normal University, Changsha, China; ^2^Medical School, Hunan University of Chinese Medicine, Changsha, China

**Keywords:** miRNA, target sites, genome, DNA, affinity precipitation

## Abstract

Combination with genomic DNA is one of the important ways for microRNAs (miRNAs) to perform biological processes. However, because of lack of an experimental method, the identified genomic sites targeted by microRNA were only located in the promoter and enhancer regions. In this study, based on affinity purification of labeled biotin at the 3′-end of miRNAs, we established an efficiently experimental method to screen miRNA binding sequences in the whole genomic regions *in vivo*. Biotinylated miR-373 was used to test our approach in MCF-7 cells, and then Sanger and next-generation sequencing were used to screen miR-373 binding sequences. Our results demonstrated that the genomic fragments precipitated by miR-373 were located not only in promoter but also in intron, exon, and intergenic. Eleven potentially miR-373 targeting genes were selected for further study, and all of these genes were significantly regulated by miR-373. Furthermore, the targeting sequences located in E-cadherin, cold-shock domain-containing protein C2 (CSDC2), and PDE4D genes could interact with miR-373 in MCF-7 cells rather than HeLa cells, which is consistent with our data that these three genes can be regulated by miR-373 in MCF-7 cells while not in HeLa cells. On the whole, this is an efficient method to identify miRNA targeting sequences in the whole genome.

## Introduction

MicroRNAs (miRNAs) are a class of endogenous small non-coding RNAs that are processed from pre-miRNAs by Dicer into 21- to 25-nt double-stranded sequences (Bartel, 2004; He and Hannon, 2004). Through regulating gene expression at the post-transcriptional level, miRNAs can take part in many biological processes including development, cell proliferation, apoptosis, organogenesis, and tumorigenesis (Carrington and Ambros, 2003; Bartel, 2004; Filipowicz et al., 2008). It has been shown clearly that miRNAs regulate gene expression on the post-transcriptional level *via* RNA-induced silencing complex (RISC) pathway in the cytoplasm (Liu et al., 2005a; Dong et al., 2013). However, with the development of new techniques, numerous miRNAs were found enriched in nucleus, which suggests that microRNAs play important roles in nucleus (Liu et al., 2018). Several studies have proved that miRNA can regulate gene expression *via* interacting with genomic sequences. In 2008, Place et al. reported that miR-373 can up-regulate cold-shock domain-containing protein C2 (CSDC2) and E-cadherin *via* sequence complementarity with the promoter of these genes. MiR-223 can combine with the promoter of NF1A and down-regulates the expression of NF1A (Place et al., 2008).

Based on the existing rule of interaction between miRNA and mRNA, some software tools for predicting miRNA binding sites in genome are developed. However, it is hard to accurately predict microRNA target sites in genome, for the mechanism of gene regulation by miRNAs *via* combination with genomic DNA remains to be elucidated. First, the location of miRNA binding sites in genome should be further studied. Janowski et al. found that small dsRNAs, which are completely complimentary with the sequence in the region −56 to +17 of the promoter, can up-regulate the expression of genes (Janowski et al., 2007). Then Meng et al. reported that the siRNA binding position can be located around −1611 from the transcription start site (Meng et al., 2016). Moreover, it was also reported that miRNA can bind in the enhancer region and increase the transcription activity of neighboring genes (Xiao et al., 2017). Second, the mechanism of interaction between genome and miRNA has not been fully illustrated. Some papers suggested that the 2–8 nt from the 5′-end of the antisense is the key to transcription activation (Xiao et al., 2017). However, it is also reported that let-7i can interact with promoter TATA-box motifs of interleukin (IL)-2 because of low minimal free energy (MFE) value (−27.6 kcal/mol), while the “seed region” of let-7i is not completely complementary with IL-2 promoter, which suggests that the complement of 5′-end of miRNA with target sequence is not the only principle for microRNA target prediction (Zhang et al., 2014). Finally, the prediction based on bioinformatics is insufficient to reflect the real condition *in vivo*, for the epigenetic modification of genome may affect the interaction of miRNA with targeting site (Liu et al., 2018).

**Figure 1:**
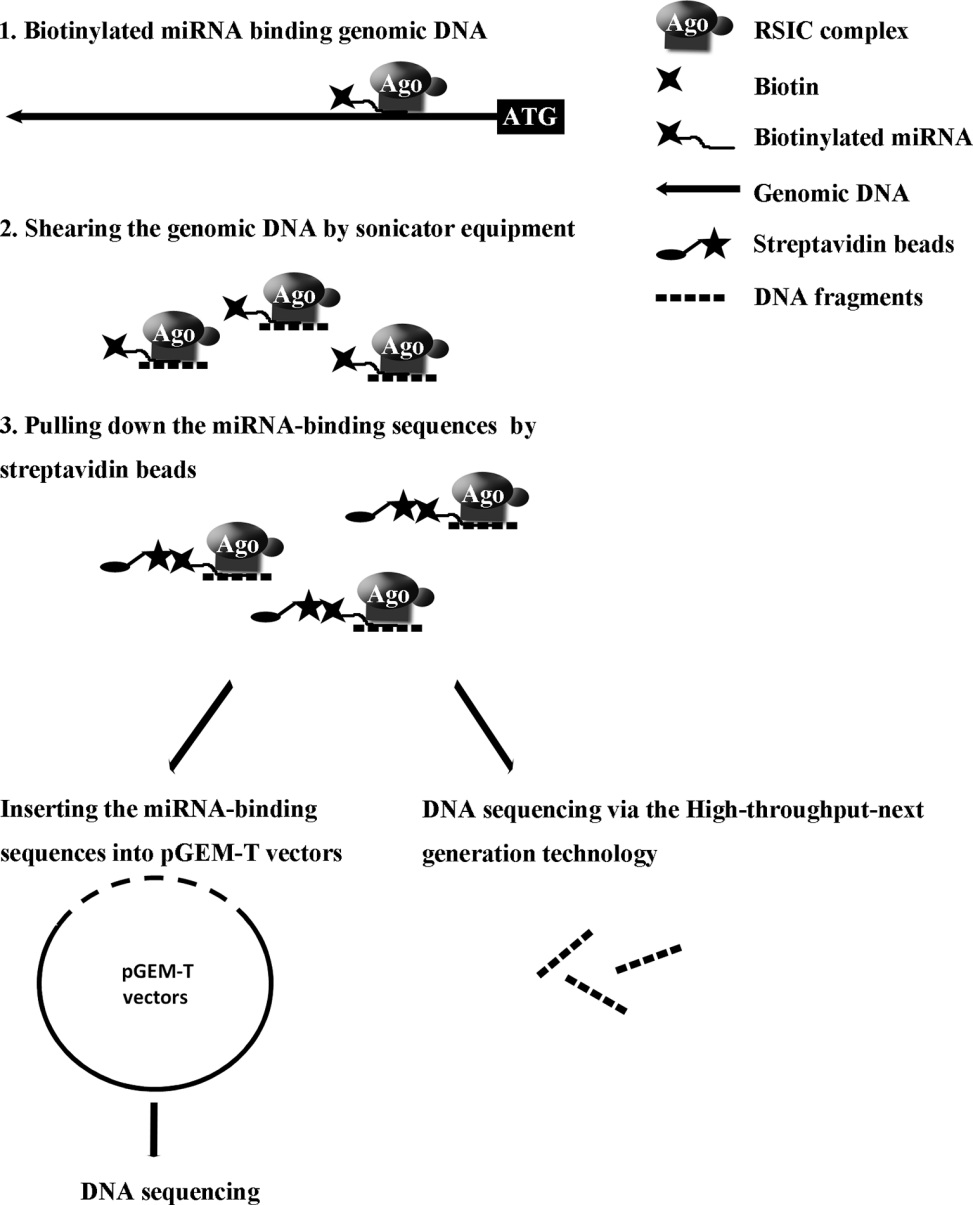
Strategy for screening microRNA binding sequences in the whole genomic regions.

Our recent study reported a convenient experimental approach for the isolation and identification of binding miRNAs for messenger RNA by applying short biotinylated DNA anti-sense oligonucleotides mix to enhanced green fluorescent protein (EGFP) mRNA, which was fused to target gene mRNA (Wei et al., 2014). We wonder whether this affinity assay could be used to screen miRNA binding sequence in genomic regions *via* biotinylated miRNA of interest. In the present study, based on biotinylated miRNA capture affinity technique, we have developed an experimental procedure for searching miRNA targeting sequences in the promoter and even in whole genomic regions (**Figure 1**). MiR-373 has been used to test our method in MCF-7. First, we proved that biotinylated miR-373, with the same function as miR-373, can up-regulate the expression of E-cadherin, which have been reported to be up-regulated by miR-373 *via* targeting its promoter. Then using the method as described in this paper, we have collected DNA fragments precipitated by biotinylated miR-373 or negative control RNA. Semi-quantitative polymerase chain reaction (PCR) and real-time PCR showed the E-cadherin promoter and CSDC2 promoter, in the previously reported miR-373 binding site, can be pulled down by biotinylated miR-373 rather than negative control RNA, which suggests that our approach is feasible. Then to find the unknown miR-373 binding sequence, the DNA fragments were inserted into pGEM-T vectors (Promega) and sequenced. Ten unreported miR-373 binding sequences were identified. Interestingly, six identified sequences were located in intron of genes and two sequences in intergenic. Only two of the rest sequences were in the promoter of gene. Western blot and real-time PCR demonstrated that six of seven identified genes can be up-regulated by miR-373 in MCF-7. Interestingly, our results shown that miR-373 cannot improve the expression of E-cadherin, CSDC2, and PDE4D in HeLa cells, which is consistent with our data that miR-373 targeting sequences of these genes cannot be precipitated by miR-373 in HeLa cells. Finally, to efficiently screen miRNA targeting genomic sequence, next-generation sequencing experiment was used to detect the samples precipitated by miR-373 and numerous miR-373 targeting sites were sequenced. On the whole, we developed an efficient approach to screen miRNA targeting genomic sequence and provided a new perspective for studying the interaction of miRNA and genome.

## Materials and Methods

### Materials

The biotinylated miR-373 and biotinylated negative control miRNA (biotinylated NC miRNA) were synthesized from TaKaRa Biotechnology (Dalian, China) *via* labeling with biotin at the 3′-end of the miRNA (**Table 1**). pGEM-T vectors were purchased from Promega (Madison, USA). ARID2, SUN1, E-cadherin, and ZNF76 antibodies were purchased from ABclonal, Inc. (Wuhan, China).

**Table 1:** miRNA sequences and real-time quantitative PCR primers.

Name		Sequence (5′–3′)
miR-373	Sense	ACUCAAAAUGGGGGCGCUUUCC
miR-373	Antisense	GAAGUGCUUCGAUUUUGGGGUGU-biotin
miRNA-NC	Sense	UCACAACCUCCUAGAAAGAGUAGA
miRNA-NC	Antisense	UCUACUCUUUCUAGGAGGUUGUGA-biotin
ARID2	Forward	ACACAGTGGTACCAGGACAG
ARID2	Reverse	TGAAGTTTGCACAGGTTGGG
PDE4D	Forward	GACCAATGTCTCAGATCAGTGG
PDE4D	Reverse	GTCAAGGGCCGGTTACCAG
E-cadherin	Forward	CCTGGGACTCCACCTACAGA
E-cadherin	Reverse	GGATGACACAGCGTGAGAGA
ZNF76	Forward	CAGGTGACGGTACAGAAAGAAGC
ZNF76	Reverse	TGATGAGCGGTGGTGTAGAGAC
SUN1	Forward	CAGAAGCACAAACAAATC
SUN1	Reverse	CACCATCATCATCAAGAC
ZNF385B	Forward	GGGCTAACCTGGAAACCGA
ZNF385B	Reverse	TCAGCTGACAGGAATTTGGACA
ALOX5	Forward	TCATCGTGGACTTTGAGCTG
ALOX5	Reverse	AGAAGGTGGGTGATGGTCTG
KIAA1958	Forward	TAGCCCTTTCTCCCTCA
KIAA1958	Reverse	TGCTCTTTCATCCTGGTC
CSDC2	Forward	GGCCCACCACATAAAATCTG
CSDC2	Reverse	ACCAACAAGCCCTCTCTCAA
β-Actin	Forward	CGTGGACATCCGCAAAGAC
β-Actin	Reverse	TCGTCATACTCCTGCTTGCTG
TTC34	Forward	TCAAAATCGACTCAGGGCAAC
TTC34	Reverse	CAGGGCTTTTTCCAGGTGG
TPM1	Forward	TTGAGAGTCGAGCCCAAAAAG
TPM1	Reverse	CATATTTGCGGTCGGCATCTT
KIAA1377	Forward	GGGCACTGAATCATCGGACAA
KIAA1377	Reverse	TTTACGTGCTCGATTTCGACA
EVI5	Forward	AGAAACCCTAGTGGGAAACAGG
EVI5	Reverse	TGACTGTATGCGATACTGTGTTC
RPL37	Forward	TCGCAATAAGACGCACACGTT
RPL37	Reverse	CTCATTCGACCAGTTCCGGT
FANCC	Forward	CTGCCATATTCCGGGTTGTTG
FANCC	Reverse	AGCACTGCGTAAACACCTGAA
ITSN2	Forward	ATACGGTGGCGGCTTGAGTT
ITSN2	Reverse	GGAAGGTGGGAAGGAGGTTGA
Hsp60	Forward	GCCACGCGGGACTCACCATT
Hsp60	Reverse	CGCTCGGTTCCAGAACTTTCCA
GAPDH	Forward	CTCACCCTGCCCTCAATATCCC
GAPDH	Reverse	AGCCCTGTAGCCTGGACCTGAT

### Cell Culture and miRNA Transfection

MCF-7 and HeLa cells were purchased from the Cell Bank of the Chinese Academy of Sciences (Shanghai, China) and cultured in Dulbecco’s modiﬁed Eagle’s medium (DMEM) (Gibco-BRL, Carlsbad, USA) supplemented with glutamine, antibiotics, and 10% fetal bovine serum (Gibco-BRL, Carlsbad, USA) in a humidified atmosphere of 5% CO_2_ at 37°C. Plasmid DNA or miRNA was transfected into cells using Lipofectamine 2000 (Invitrogen, Carlsbad, CA) according to the manufacturer’s instructions.

### Western Blot

Cells were harvested 24, 48, or 72 h post-transfection. Then cells were lysed in radioimmunoprecipitation assay (RIPA) buffer [150-mM NaCl, 1-M Tris-HCl (pH 7.2), 1% (v/v) Triton X-100, 1% (w/v) sodium deoxycholate, 0.1% (w/v) sodium dodecyl sulfate (SDS)] with protease inhibitors. Proteins were separated on 10% or 15% SDS–polyacrylamide gel and transferred to poly(vinylidene diﬂuoride) (PVDF) membranes. The resulting blots were blocked with 5% non-fat dry milk, and specific proteins were detected with appropriate antibodies. The proteins were detected using horseradish peroxidase (HRP)-conjugated secondary antibody and Super Signal West Pico Chemiluminescent substrate kits (Pierce).

### DNA–miRNA Pull-Down Assay

The procedures used for affinity purification of biotinylated miRNAs were partly in reference to those previously described by Tidi Hassan and colleagues (Liu et al., 2005b). Cells were transfected by biotinylated miRNA or biotinylated NC miRNA for 24 h. Then cells were treated with 37% formaldehyde to a final concentration of 1% and incubated at room temperature for 15 min for cross-linking. The cross-linking reaction was stopped by the addition of 100-mM glycine. Next, cells were collected; lysed in lysis buffer that contains 1% SDS, 1-mM EDTA, 50-mM HEPES (pH 7.5), 140-mM NaCl, and 1% Triton X-100; and supplemented with 100× protease inhibitor (Boehringer cocktail) and 1-U/μl RNase inhibitor (Invitrogen). The genomic DNA was sheared by sonicator equipment. This step should be performed on ice to avoid the denaturation of chromatin and miRNA. The supernatants were recovered by 12,000*g* centrifugation for 10 min and incubated with equilibrium streptavidin beads for 1 h at room temperature. Streptavidin beads were washed four times by washing buffer, which contains 10-mM Tris-HCl (pH 7.5), 1-mM EDTA, 0.15-mM LiCl, and 10-mM Tris-HCl. Proteinase K (Roche Applied Science) and RNase A (Roche Applied Science) were be used to degrade protein and RNA. Then the DNA was separated from streptavidin beads after treating beads at 80°C for 5 min. The eluted DNA was recovered using Chromatin Immunoprecipitation (ChIP) Kit (Millipore, USA) according to manufacturer’s instructions.

### Illumina HiSeq 2000 Next-Generation Sequencing and Bioinformatics Analysis

The PCR products were fragmented to an average length of 150 bp. After DNA-end repair, 3′dA overhang, and ligation of methylated sequencing have been performed, the DNA samples were sent to Beijing Genomics Institute (BGI, China) for sequencing by Illumina Genome Analyzer. Bioinformatics analysis steps for ChIP-Seq libraries are presented below. First, the original image data are transferred into sequence data *via* base calling, which is defined as raw data or raw reads, and saved as FASTQ file. Second, quality control was performed to detect whether the data are qualified. In addition, filtering of raw data was used to decrease data noise. As a result, “dirty” raw reads which contain the sequence of adapter, more than 10% unknown bases, or low-quality bases have been removed in this step. Third, the clean reads were mapped to the *Homo sapiens* genome reference, and only the alignments within two mismatches and unique mapping reads were considered in further analyses. Then genome-wide peak scanning was performed in UCSC Genome Browser to get the information of peak location and peak sequence. Peaks were classified based on the location (UCSC annotation data) and showed in the following genome regions: intergenic, introns, downstream, upstream, and exons. Furthermore, after peak scanning, all the related genes related to miR-373 or NC RNA can be listed. Last, to predict potential functions of the putative miRNA targets in different cellular components, biological processes, and molecular functions, we used gene ontology (GO) categories (http://www.geneontology.org/) to classify the identified target genes. Besides, the Kyoto Encyclopedia of Genes and Genomes (KEGG) database (fttp://fttp.genome.jp/pub/kegg/pathway/) was applied for KEGG pathway analyses. We also submitted the HiSeq 2000 next-generation sequencing data to National Center for Biotechnology Information (NCBI).

### Validation of miRNA Targets *via* qRT–PCR

Total RNA was isolated from cells that were transfected with synthetic miRNAs using TRIzol reagent (TaKaRa) according to the manufacturer’s instructions. For quantification of mRNA, 1 µg of total RNA was reversely transcribed using the Reverse Transcription System (Promega, Madison, USA). The resulting cDNA was used as template for semi-quantitative PCR or quantitative real-time PCR. β-Actin served as an endogenous control used to normalized expression data. Each sample was analyzed in triplicate. Relative expression and standard error were calculated by the supplied ABI 7900HT Real-Time System software. All primers used in the qRT–PCR experiments are listed in **Table 1**.

### Statistical Analysis

Data were expressed as means ± SD from three to four independent experiments. Data were analyzed using Student’s *t* test for two groups or analysis of variance (ANOVA) with Tukey–Kramer tests for multiple group comparisons. *P* < 0.05 was considered statistically significant.

## Results

### E-Cadherin Is Up-Regulated by Both miR-373 and Biotinylated miR-373 in MCF-7

**Figure 2:**
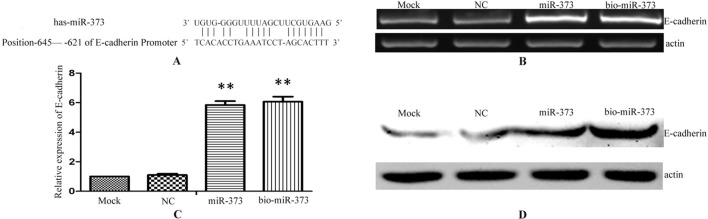
Biotinylated miR-373 can regulate the expression of E-cadherin. **(A)** The identified miR-373 binding site in E-cadherin promoter. MCF-7 cells were transfected with miR-373, NC miRNA, or bio-miR-373 for 48 h. Then semi-quantitative PCR **(B)**, real-time PCR **(C)**, and Western blotting **(D)** were performed to detect the expression of E-cadherin. *n* = 3, **P < 0.01 compared with the sample transfected with NC miRNA.

Previous reports described that miR-373 can increase the expression of E-cadherin *via* targeting to its promoter in PC-3 cells (**Figure 2A**), while it has no impact on E-cadherin expression in HCT-116 and LNCaP cells (Place et al., 2008). To ensure whether miR-373 and biotinylated miR-373 could regulate E-cadherin in MCF-7 cells, biotinylated miR-373, non-biotinylated miR-373, or NC miRNA was transfected into MCF-7 cells for 48 h. Both semi-quantitative PCR and real-time PCR showed that E-cadherin increases over four times in the mRNA level after transfection with miR-373 or biotinylated miR-373 than does NC miRNA (**Figures 2B, C**). We also confirmed that both miR-373 and biotinylated miR-373 can up-regulate E-cadherin protein levels in MCF-7 cells (**Figure 2D**). These data demonstrated that miR-373 increases the expression of E-cadherin in MCF-7 cells. Furthermore, being labeled with biotin at the 3′-end of miR-373 would not significantly affect the function of miR-373 to regulate E-cadherin.

### The Promoters of E-Cadherin and CSDC2 Can be Pulled Down by Biotinylated miR-373 in MCF-7

**Figure 3:**
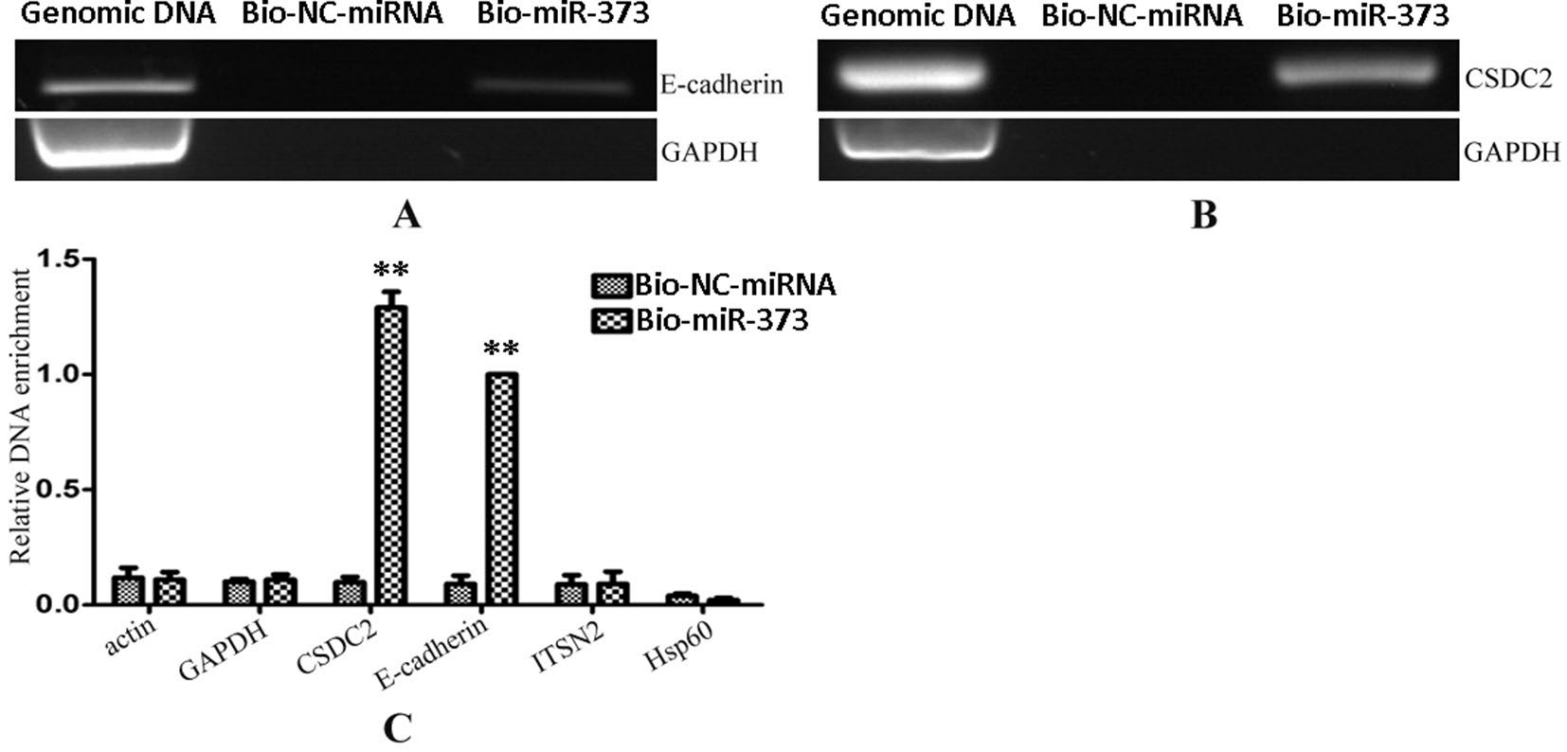
Promoters of E-cadherin and CSDC2 can be pulled down by biotinylated miR-373. Bio-miR-373 or bio-NC-miRNA was transfected into MCF-7 cells for 24 h. Then cells were harvested, and the DNA fragments were enriched *via* DNA–miRNA pull-down assay. **(A)** The enrichments of E-cadherin promoter were measured by semi-quantitative PCR. **(B)** The enrichments of CSDC2 promoter were measured by semi-quantitative PCR. **(C)** Real-time PCR was performed to detect the enrichments of E-cadherin and CSDC2 promoters. U6 fragments added in the samples were taken as an endogenous control. *n* = 3, **P < 0.01 compared with the sample transfected with bio-NC-miRNA.

After having confirmed that biotinylated miR-373 could increase the expression of E-cadherin in MCF-7, the miR-373 targeting sequence in E-cadherin promoter was regarded as a positive control to test whether our method worked. Biotinylated miR-373 or biotinylated NC miRNA was transfected into MCF-7 cells. After 24 h post-transfection, miR-373 targeting sequences were isolated through DNA–miRNA pull-down assay described in the Materials and Methods section. Then semi-quantitative PCR and quantitative RT–PCR were performed to detect the enrichment of E-cadherin and CSDC2 promoters. As shown in **Figures 3A, B**, both E-cadherin and CSDC2 promoters can be amplified by semi-quantitative PCR from the sample transfected with biotinylated miR-373 rather than biotinylated NC miRNA. GAPDH promoter, which did not contain potential target site of miR-373, has no detectable signal when the sample transfected with biotinylated miR-373 was used. As shown in **Figure 3C**, the quantitative RT–PCR results were consistent with those of semi-quantitative PCR. The amount of E-cadherin promoter and CSDC2 promoter in the sample transfected with biotinylated miR-373 was as over 10 times as the sample transfected with biotinylated NC miRNA, while the amount of promoters of actin, GAPDH, ITSN2, and Hsp60, which were regarded as negative control, is almost the same in the sample transfected with biotinylated miR-373, compared with the sample transfected with biotinylated NC miRNA. These results suggested that our method can be used to enrich miR-373 binding DNA sequences.

### Identifying the Potential miR-373 Targeting DNA Sequences in Purified DNA Products

To identify unknown miR-373 targeting DNA sequences, we inserted the precipitated DNA into pGEM-T vectors (Promega, USA) and sequenced the vectors using the primer combining with T7 promoter. The specific procedure is shown in **Figure 1**. First, the Quick Blunting Kit (NEB, USA) has been used to convert DNA with incompatible 5′ or 3′ overhangs to blunt-ended DNA, because bio-miR-373-precipitated DNA fragments sheared by sonicator equipment contain fragments with incompatible 5′ or 3′ overhangs, which were hard to insert into pGEM-T vectors. Second, we tailed the blunt-ended DNA with an adenine in the 3′-end *via* using Ex Taq DNA Polymerase. Third, to improve the efficiency to insert DNA fragments into pGEM-T vectors, the A-tailing fragments have been condensed by 20% PEG-8000 and purified by 75% alcohol. Fourth, the purified DNA fragments have been inserted into pGEM-T vectors (Promega, USA). Fifth, the vectors were transformed into Top10 (Invitrogen, USA), and blue-white screening was used to select positive clones. Over 40 clones have been sequenced, and 10 sequences have been identified. Last, the sequences were analyzed *via* UCSC Genome Browser and NCBI Map Viewer. As shown in **Table 2**, six identified sequences are located in the introns of ZNF76, PDE4D, ALOX5, KIAA1959, and ZNF385B. Two sequences are in intergenic. Two sequences are in promoters of ARID2 and SUN1.

**Table 2:** miR-373 binding sequences identified by Sanger sequencing.

BLAT location	Located gene	GenBank accession no.	Location
Chr12: 46122709-46123286	ARID2	NC_000012.11	Promoter
Chr6: 35234579-35234951	ZNF76	NC_000006.11	Intron
Chr5: 59660052-59660485	PDE4D	NC_000005.9	Intron
Chr7: 814338-814698	SUN1	NC_000007.14	Promoter
Chr2: 180706031-180706314	ZNF385B	NC_000002.11	Intron
Chr10: 45883501-45883757	ALOX5	NC_000010.10	Intron
Chr9: 115377923-115378596	KIAA1958	NC_000009.11	Intron
Chr 21: 42657214-42657541	Not found	Not found	Intergenic
Chr 10: 2979740-2980027	Not found	Not found	Intergenic

### The Regulation of the Identified Genes by miR-373

**Figure 4:**
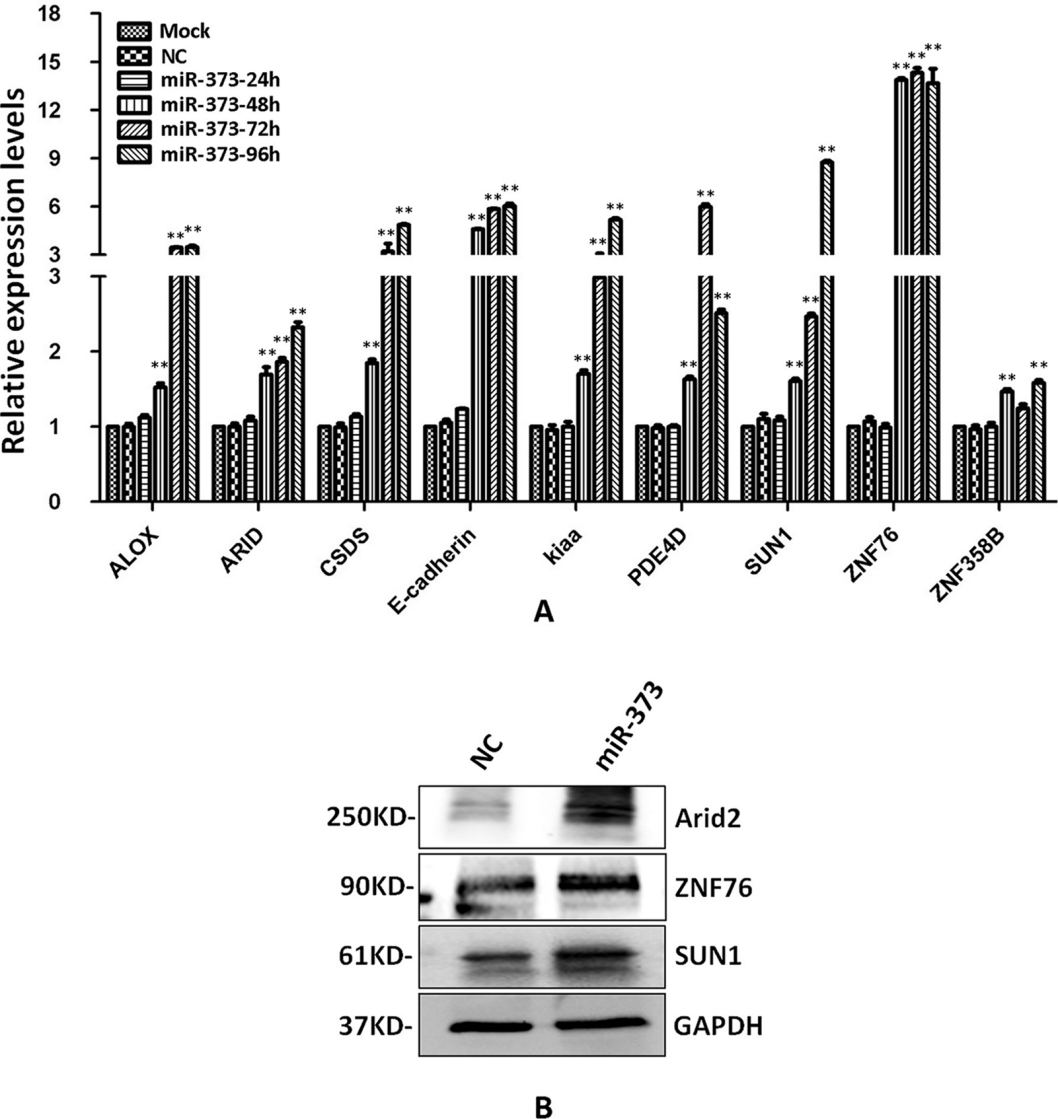
The identified genes can be regulated by miR-373. **(A)** MCF-7 cells were transfected with miR-373 or NC-miRNA. Cells were harvested after 24, 28, 72, or 96 h post-transfection. Real-time PCR was used to measure the mRNA level of identified genes. The expression of β-actin was used as the internal reference. *n* = 3, **P < 0.01 compared with the sample transfected with NC miRNA. **(B)** MiR-373 or NC-miRNA was transfected into MCF-7 cell for 72 h. The protein levels of identified genes were detected by Western blotting.

The identified miR-373 targeting sequences are not only located in promoters but also located in introns. We wonder whether miR-373 could regulate these genes *via* directly binding with its promoter or intron. First, quantitative RT–PCR and Western blotting were performed to detect the regulation of the identified genes by miR-373. As shown in **Figure 4A**, the mRNA levels were not observed to be significantly changed at 24 h post-transfection with miR-373. MiR-373 increases the mRNAs of ALOX, ARID2, CSDS2, KIAA1958, PDE4D, SUN1, and ZNF358B only 30% to 80% times at 48 h, while it up-regulates the mRNAs of E-cadherin and ZNF76 over four times at 48 h. All genes were up-regulated over two times in the mRNA level by miR-373 at 72 h, excepting ZNF358B. Then we purchased ARID2, SUN1, and ZNF76 antibodies to detect the expression of these genes at protein levels. As shown in **Figure 4B**, miR-373 can significantly increase these genes at 72 h. These data indicated that ARID2, SUN1, and ZNF76 can be obviously up-regulated by miR-373.

**Figure 5:**
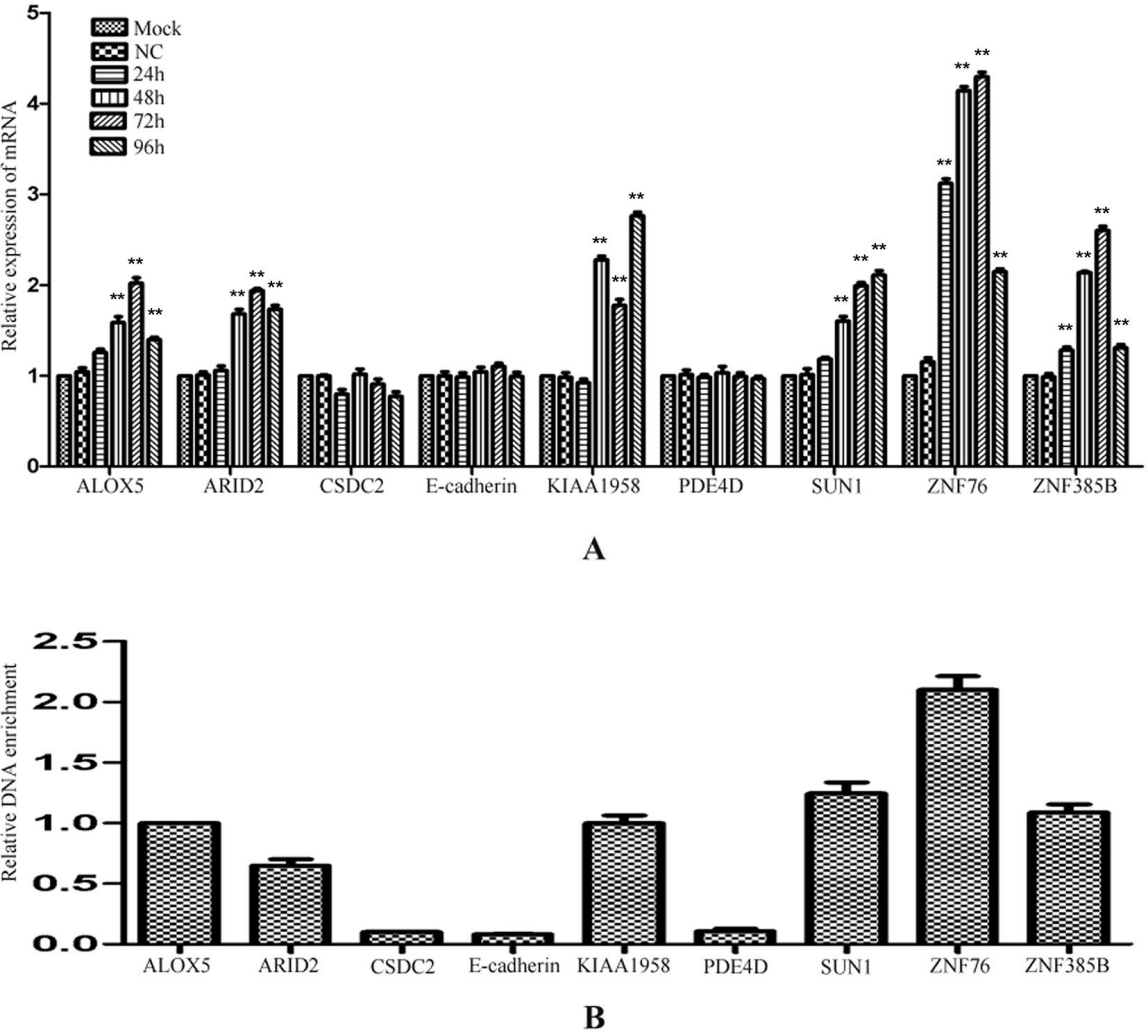
The regulation of identified genes by miR-373 in HeLa cells. **(A)** HeLa cells were transfected with miR-373 or NC-miRNA. Cells were harvested after 24, 48, 72, or 96 h post-transfection. Real-time PCR was used to measure the mRNA level of ALOX5, ARID2, E-cadherin, KIAA1958, PDE4D, SUN1, ZNF76, and ZNF385B genes. The expression of β-actin was used as the internal reference. *n* = 3, *P < 0.05, **P < 0.01 compared with the sample transfected with NC miRNA. **(B)** Bio-miR-373 or bio-NC-miRNA was transfected into HeLa cells. After 24 h post-transfection, DNA–miRNA pull-down assays were performed to enrich the miR-373 binding sequences. Then real-time PCR was performed to detect the enrichments of the genomic sequences in ALOX5, ARID2, E-cadherin, KIAA1958, PDE4D, SUN1, ZNF76, and ZNF385B genes. U6 fragments added in the samples were taken as an endogenous control.

To investigate whether the regulation by miR-373 to the identified genes is a common phenomenon, RT–PCR was performed to measure the expression of the identified genes in HeLa cells. Interestingly, ALOX5, ARID2, KIAA1958, SUN-1, ZNF76, and ZNF385B can be up-regulated by miR-373, while CSDC2, E-cadherin, and PDE4D have not significantly increased after being transfected with miR-373 (**Figure 5A**). We also found that miR-373 cannot bind with the targeting sequence in CSDC2, E-cadherin, and PDE4D genes (**Figure 5B**). Some papers reported that some cell lines were resistant to specific miRNAs or dsRNAs-reduced transcriptional activation while sensitive to others. Our results provide an evidence that the direct interaction between miRNA and genomic sequences is key to miRNA-induced regulation of genes.

### Screening miR-373 Binding Sequences *via* the High-Throughput Next-Generation Sequencing Technology

We have successfully established a method to identify unknown miRNA targeting DNA sites, but only 10 sequences have been identified in over 40 clones (data not shown). To improve efficiency to screen unknown miRNA target sequences, two genomic DNA fragment libraries were constructed and subjected to next-generation sequencing: one was constructed from miR-373-precipitated sample in MCF-7 and named HM-7-DNA, and the other was from NC RNA-precipitated sample and named HM-7-DNA-NC. As shown in **Supplementary Figure 1**, the main peak of HM-DNA sample was distributed at 360 bp and the main peak of HM-DNA-NC sample was distributed at 276 bp. So both of the samples were qualified and suitable for further sequencing. Then the quality control was used to analyze the quality of raw data obtained from Illumina HiSeq 2000 sequencing. As shown in **Supplementary Figures 1A, C**, both HM-7-DNA and HM-7-DNA-NC represented good-quality sequences, because the base ratios are mostly higher than 20. The raw data also had satisfactory base composition, for four bases of A, T, G, and C were distributed uniformly, and the AT content exceeded the GC content (**Supplementary Figures 2B, D**). The raw data have been submitted into SRA database, and the accession number is PRJNA547356.

**Figure 6:**
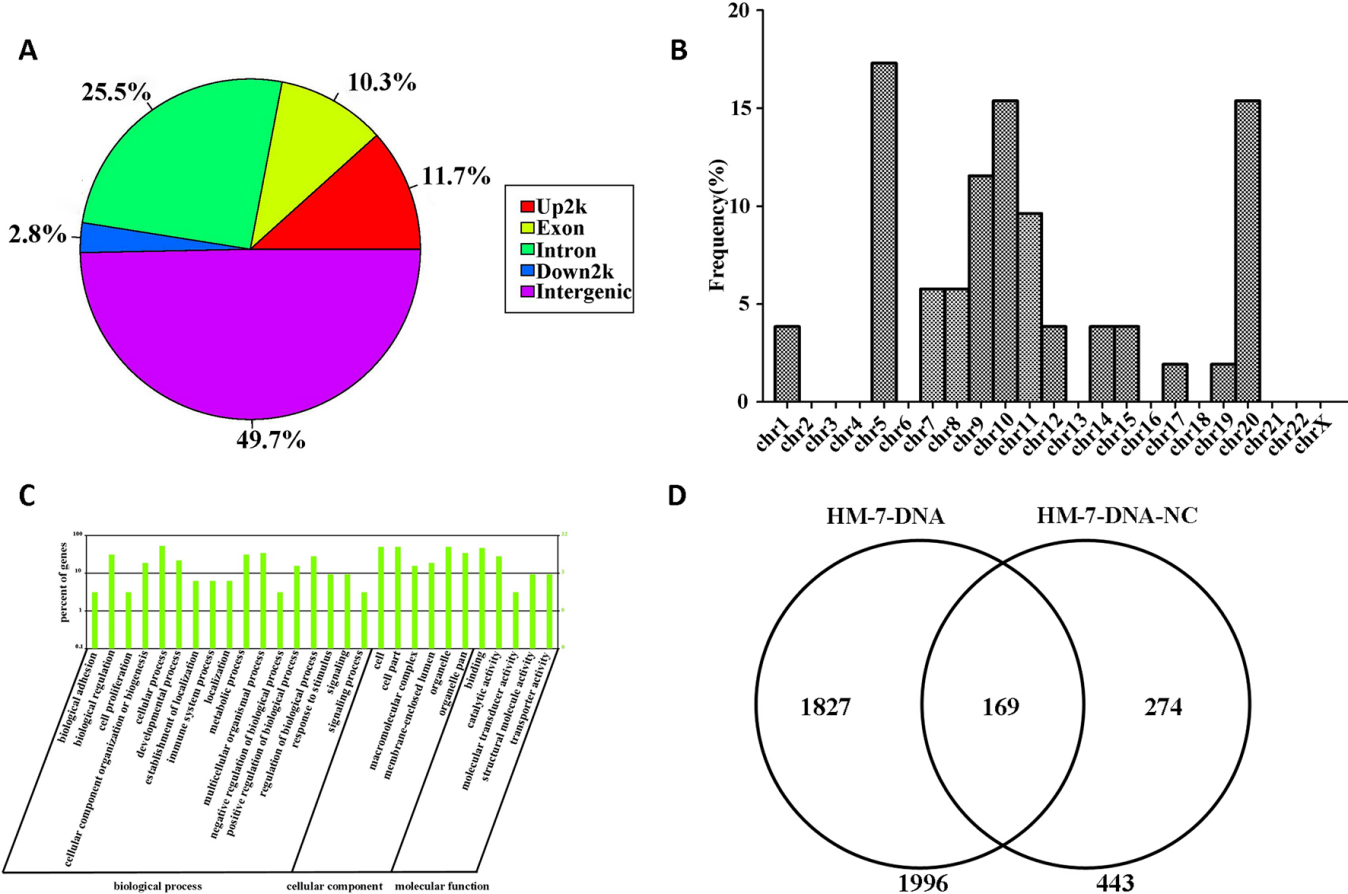
Screening genomic sequences by next-generation sequencing. **(A)** The distribution of functional elements of DNA sequences in HM-7-DNA. **(B)** The chromosomal location of potential target genes in HM-7-DNA. **(C)** Gene ontology analysis of peak-related gene of HM-7-DNA. **(D)** Differential analysis of the potential target genes of HM-7-DNA and HM-7-DNA-NC.

The information of the peak location and sequence has been identified by genome-wide peak scanning in UCSC Genome Browser (**Supplementary Tables 1**, **2**). Then we analyzed the distribution of the sequences from HM-7-DNA. As shown in **Figure 6A**, 49.7% of the sequences are located in intergenic, 25.5% of the sequences in intron, 11.7% of the sequences in promoter (Up2k), 10.3% of the sequences in exon, and 2.8% of the sequences in down2k. Meanwhile, we also analyzed the chromosomal location of miR-373 targeting sequences (**Figure 6B**). The results showed that the candidate targets of miR-373 were mainly distributed in 5th, 9th, 10th, and 20th chromosomes (**Figure 6B**).

MiRNAs can perform its biological functions *via* targeting genomic DNA and regulating gene expression, so pathway-based analysis of miR-373 targeting gene helps us to better understand the role of miR-373 in cells. On the one hand, GO analysis was performed to annotate the function of genes. **Figure 6C** shows the classification of the peak-related gene of HM-7-DNA based on the GO analysis. Biological process, cellular component, and molecular function, respectively, included 17, 6, and 5 categories. On the other hand, based on KEGG analysis, we found that miR-373 targeting genes were related with hypertrophic cardiomyopathy, dilated cardiomyopathy, tight junction, cardiac muscle contraction, and viral myocarditis (**Table 3**).

**Table 3:** The significant enrichment analysis of target gene’s function in the pathway.

Pathway ID	Pathway	*Q* value
ko05410	Hypertrophic cardiomyopathy (HCM)	0.001193353
ko05414	Dilated cardiomyopathy	0.001193353
ko04530	Tight junction	0.001193353
ko04260	Cardiac muscle contraction	0.001361812
ko05416	Viral myocarditis	0.019827399

Q ≤ 0.05 is a pathway that was significantly enriched in the peak-related gene.

We also compared the differences between HM-7-DNA and HM-7-DNA-NC. As shown in **Figure 6D**, 1,966 genes containing miR-373 targeting sequences have been found. Interestingly, 443 genes containing NC miRNA targeting sequences also have been identified. It cannot be denied that all designed NC miRNAs have the ability to combine with certain DNAs, so it is a possibility to have NC miRNA binding sites in genomic DNA. According to our results, there are 169 genes containing both miR-373 and NC miRNA target sites. These results suggest that the NC miRNA used in our paper is not suitable for studying the regulation of these 169 genes by miR-373, because NC miRNA also has a possibility to regulate these genes. Hence, to better study the genes identified by our method, it is necessary to use biotinylated NC miRNA as negative control to prove that the studied genes have no potential NC miRNA binding sites.

**Figure 7:**
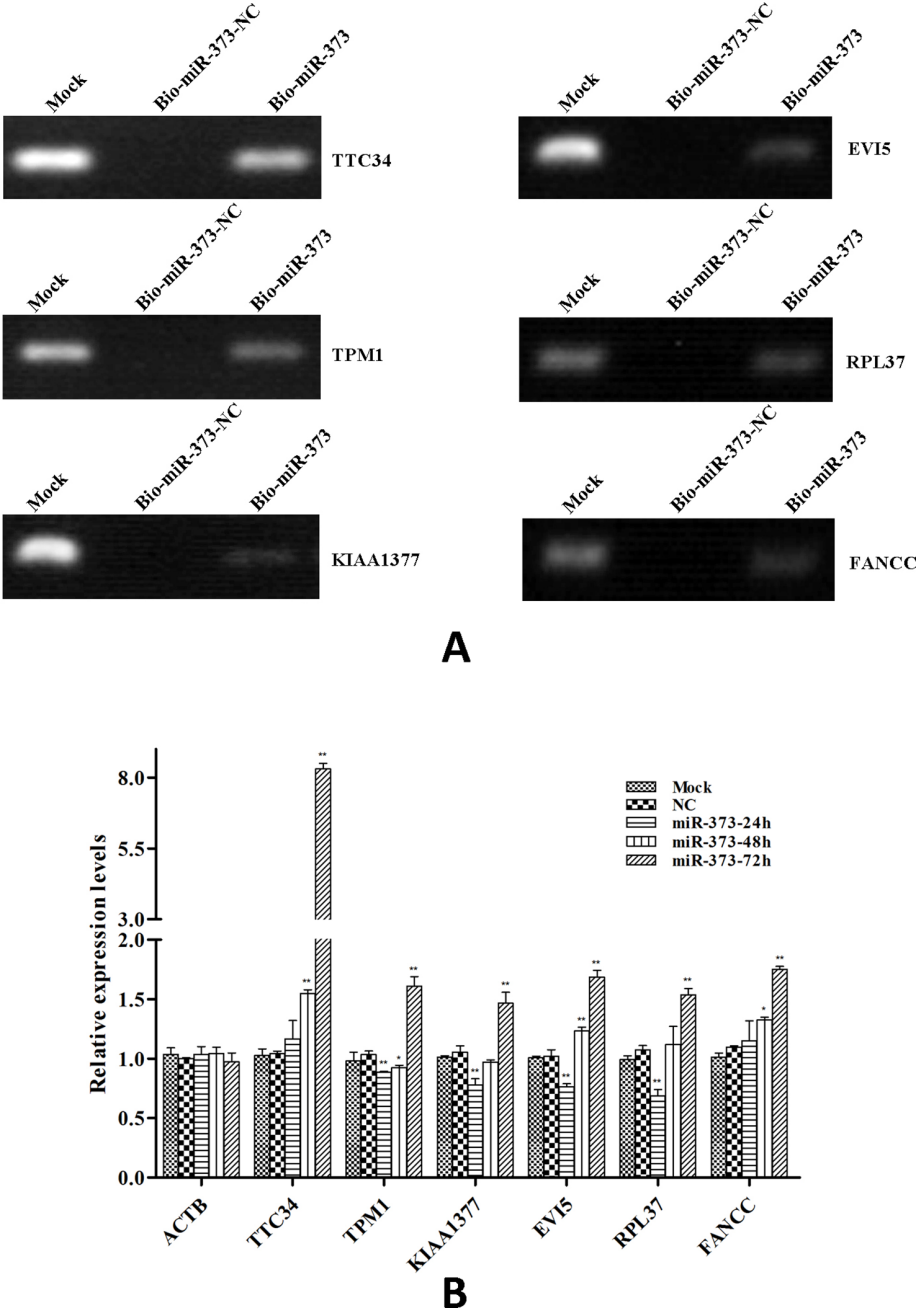
The identified genes *via* next-generation sequencing can be regulated by miR-373. **(A)** Bio-miR-373 or bio-NC-miRNA was transfected into MCF-7 cells for 24 h. Then cells were harvested, and the DNA fragments were enriched *via* DNA–miRNA pull-down assay. The enrichments of TTC34, EVI5, TPM1, RPL37, KIAA1377, and FANCC promoters were measured by semi-quantitative PCR. **(B)** MCF-7 cells were transfected with miR-373 or NC-miRNA. Cells were harvested after 24, 48, or 72 h post-transfection. Real-time PCR was used to measure the mRNA level of identified genes. The expression of β-actin was used as the internal reference. *n* = 3, *P < 0.05, **P < 0.01 compared with the sample transfected with NC miRNA.

So we selected six sequences from 1,827 genes that only contain miR-373 targeting sites to do further study. These sequences are located in exon or intron (**Table 4**). Semi-quantitative PCR has been performed to detect the enrichment of the six sequences in miR-373-precipitated DNA. As shown in **Figure 7A**, all sequences can be pulled down by miR-373. Then quantitative RT–PCR results demonstrated that the mRNAs of TTC34 and FANCC significantly increase after transfection by miR-373 for 48 and 72 h. TPM1, KIAA1377, EVI15, and RPL37 can be down-regulated by miR-373 after 24 h post-transfection, while these genes can be significantly up-regulated by miR-373 at 72 h (**Figure 7B**). We also randomly selected 35 potential miR-373 target genes and analyzed the changes in these gene expression after transfecting miR-373 for 48 h. As shown in **Table 5**, 21 gene expression changed more than two-fold and only four gene expression changed less than quarter-fold after transfecting with miR-373 than did NC miRNA.

**Table 4:** MiR-373 binding sequences identified by next-generation sequencing.

BLAT location	Located gene	GenBank accession no.	Location
Chr1: 2585008-2585961	TTC34	NC_000001.10	Intron
Chr15: 63335003-63335190	TPM1	NC_000015.9	Exon
Chr11: 101785594-101785989	KIAA1377	NC_000011.9	Intron/exon
Chr1: 93143762-93143908	EVI5	NC_000001.10	Intron
Chr5: 40835156-40835380	RPL37	NC_000005.9	Intron/promoter
Chr9: 97937920-97938060	FANCC	NC_000009.11	Intron

**Table 5:** The identified genes affected by miR-373.

Gene name	Gene ID	Fold change
RPL37A	6168	−2.98
tnni3	7137	−5.34
KATNAL2	83473	−2.35
DOCK8	81704	1.85
COL23A1	91522	1.83
TNNT2	7139	4.03
GPR75	10936	−1.89
ASB3	51130	1.22
ATP5G3	518	−1.33
IVNS1ABP	10625	−4.81
WASH3P	374666	1.58
ARMC9	80210	−1.03
BAIAP2L1	55971	2.02
CRYAB	1410	2.73
NBAS	51594	1.61
GALNT9	50614	1.66
TMOD2	29767	−4.03
mapk6	5597	−3.86
Ybx1	4904	−1.67
rpl13	23521	−2.81
ralgps2	55103	−3.71
ITGB3BP	23421	−9.89
RHBDL2	54933	−3.31
PRMT8	56341	−2.38
COX11P1	140468	−6.28
DNAH14	127602	2.32
HEATR1	55127	−4.05
R3HCC1L	27291	−2.73
TMEM99	147184	1.08
ENKUR	219670	−5.13
WIPF1	7456	−1.44
SPTSSB	165679	−9.34
LGR4	55366	−2.28
LTBP1	4052	−1.33
LRCH3	84859	−1.22

## Discussion

Although it has been proved that binding with promoters is an important way for miRNA to regulate gene expression, the mechanism of miRNA target recognition in genome should be further illuminated. Similar to the miRNA–mRNA interaction model, some papers suggested the “seed sequence” in miRNA is the key to binding with promoter (Xu et al., 2014; Xiao et al., 2017). However, Zhang et al. reported that let-7i can bind with TATA-box motifs in IL-2 promoter and the seed sequence of let-7i is not completely complementary with IL-2 promoter (Zhang et al., 2014). Another type of prediction tools, such as RNA hybrid, evaluates the interaction ability between miRNA and genomic sequences *via* measuring thermo-dynamic stability of miRNA and dsDNA or ssDNA (Rehmsmeier et al., 2004). Furthermore, Paugh et al. proved that miRNAs can form triplexes with dsDNA in genome and regulate gene expression (Paugh et al., 2016). Because of lacking support by mechanism, computational prediction of miRNA targeting site in genome is in an initial step. In this paper, based on miRNA targeting–mRNA purification technique, which has been reported previously (Hassan et al., 2013), we have established an effective biochemical procedure to screen the potential miRNA targeting genes *via* pulling down the genomic sequences, which directly combined by miRNA. As a result, the putative target DNA sequences that were bound by biotinylated miRNAs can be easily isolated from cell extracts. These isolated DNA sequences can be analyzed through cloning and sequencing, and then the potential target genes may be found using the bioinformatics analysis. As described in this article, we successfully identified the known target genes of miR-373; moreover, we also detected unreported target genes. Therefore, we demonstrated that the target genes of miRNA complementary to DNA sequences can be efficiently obtained through our biochemical procedure directly from cultured cells.

Another limitation of bioinformatic prediction is that the prediction cannot reflect the real situation *in vivo*. When Li et al. have studied the regulation of gene expression by dsRNA *via* binding with promoter, they found that some specific dsRNAs can increase target gene expression in some cell lines, but not in others (Li et al., 2006). They also reported that E-cadherin expression was up-regulated by miR-373 in PC-3 and LNCaP cells, while not in HCT-116 cells (Place et al., 2008). Meanwhile, our results demonstrated that the expression of E-cadherin, CSCD2, and PDE4D, which can be up-regulated by miR-373 in MCF-7 cells, was not significantly increased after transfecting miR-373 in HeLa cells. One of the reasons affecting miRNA-mediated gene activation is the epigenetic state of genome, for it is proved that the promoter of E-Cadherin is hypermethylated in HeLa cells, which prevented saRNA-induced E-cadherin up-regulation (Li et al., 2006). Furthermore, our results demonstrated miR-373 can interact with the sequences located in E-cadherin, CSCD2, and PDE4D genes in MCF-7 cells but cannot in HeLa cells, which indicated that the direct interaction of miRNAs and targeting sequences is key to regulation of targeting genes. On the whole, our method can measure the direct interaction between miRNA and genomic DNA, which can avoid false positives caused by ignoring the modification of genome.

A very noteworthy finding in the present study is that some genomic fragments precipitated by miR-373 were located in intron. The roles of miRNAs in intron have not been widely studied. Meng et al. reported that some miRNAs binding sites are located in intron in plants (Meng et al., 2013). It has also been reported that siRNA targeting intronic sequences near alternative exons regulate splicing of mRNA and that Ago1 is essential for RNAi-mediated alternative splicing (Allo et al., 2009). It has been reported that some miRNAs and long noncoding RNAs are transcribed from the intron *via* sharing of the promoters with their host genes (Bosia et al., 2012; Kung et al., 2013; Chamorro-Jorganes et al., 2014; Ramalingam et al., 2014), so miRNAs (e.g., miR-373) targeting intron may play a role in regulating miRNAs and long noncoding RNAs, which are located in intron. Our results demonstrated that miR-373 can interact with the sequences located in intron. Then our results showed that ZNF76, PDE4D, ALOX5, KIAA1958, ZNF385B, TTC34, EVI5, and FANCC, which contain miR-373 binding sites in intron, can be regulated by miR-373. So there is existing interaction among miR-373 and intron sequences, which might affect gene expression. Taken together, the results suggested that the interaction of miRNAs and intron may play some biological functions in cells, though we have not provided direct evidence that miRNAs regulate gene expression *via* binding with intron. The expression level of ZNF76 mRNA regulated by miR-373 was dramatic increased, but this regulated mechanism need to study in further study should focus on the mechanism of ZNF76 regulation by miR-373. We will investigate whether miR-373 binding sequence in ZNF76 intron is key to regulate expression of ZNF76 by knocking out the sequence.

Although we successfully identified several miR-373 binding sequences located in promoter, exon, or intron, there were some purified DNA fragments located in genomic DNA region far away from any known gene (over 80 kb, data not show). These DNA fragments may be located near the uncharacterized genes or may be a trigger for mediating the long-range regulation as described in a previous report (Zhao et al., 2009).The biological function of interaction between miRNAs and these target sites need to be further researched. However, our experimental procedure provides a way to find this kind of target sites and uncover new regulated mechanism of miRNAs.

Strangely, there is lack of connection between Sanger sequencing and next-generation sequencing results. Because Sanger sequencing and next-generation sequencing results were obtained from two independent experiments, many factors, including cell culture conditions, ChIP, and library construction, may contribute to variability between datasets. Another reason that may contribute to variability between two kinds of sequencing is that the samples have been prepared *via* different procedures. Preparing the samples for next-generation sequencing, compared with the samples for Sanger sequencing, have an extra step in that samples need to be amplified by PCR. In this step, some CG-rich sequences may be lost because the sequences are hard to be amplified by PCR.

In conclusion, this is a suitable method for identifying miRNA target genes that are complementary to genomic DNA. With the use of this method, the interaction of miRNA and putative miRNA targets can be confirmed by quantitative PCR with specific primers. So this method can be used to confirm the regulation mechanism of miRNAs to genes *via* binding genomic DNA. Furthermore, the experimental procedure can be applied to screen potential miRNA targets. On the whole, the method can improve the miRNA research enormously.

## Data Availability

The raw data was submitted to NCBI SRA database and the accession number is PRJNA547356.

## Author Contributions

Designed the experiments: SX, KW. Performed the experiments: YX, KW, YT, LH, HX, SL, MG, CW. Wrote the paper: YX, KW, SX.

## Funding

This research was funded by the China Natural Science Foundation (grant numbers 81770389, 81601122, and 81703919), Hunan Provincial Natural Science Foundation of China (grant number 2017JJ3205, 2017JJ3232), and Cooperative Innovation Center of Engineering and New Products for Developmental Biology of Hunan Province (grant number 20134486).

## Conflict of Interest Statement

The authors declare that the research was conducted in the absence of any commercial or financial relationships that could be construed as a potential conflict of interest.

## Abbreviations

CSDC2, cold-shock domain-containing C2; ITSN2,intersectin 2; Hsp60, heat shock protein family D (Hsp60) member 1; ALOX5, arachidonate 5-lipoxygenase; ARID2, AT-rich interaction domain 2; PDE4D, phosphodiesterase 4D; SUN1, Sad1 and UNC84 domain-containing 1; ZNF76, zinc finger protein 76; ZNF385B, ZNF385B; TTC34, tetratricopeptide repeat domain 34; TPM1, tropomyosin 1; EVI5, ecotropic viral integration site 5; RPL37, ribosomal protein L37; FANCC, FA complementation group C.

## Supplementary Material

Supplementary Figure 1 Distribution of the main peak of genomic DNA. The genomic DNA was sheared by sonicator equipment. Then 1.5% agarose gel was used to measure the quality of samples. Results from **(A)** HM-DNA and **(B)** HM-DNA-NC are; both the main peaks of genomic DNAs were distributed between 100 and 500 bp. Agilent 2100 was used to detect specific distribution of sample fragments. HM-DNA fragment distributed at 360 bp **(C)**; HM-DNA-NC fragment distributed at 276 bp **(D)**.

Supplementary Figure 2 Quality distribution and base distribution of HM-7-DNA and HM-DNA-NC. Quality distribution of **(A)** HM-7-DNA and **(C)** HM-7-DNA-NC are shown; the *X*-axis corresponds to the base site of the read. The *Y*-axis is quality value. Each dot in the image represents the quality value of the corresponding position along reads. Base distribution of **(B)** HM-7-DNA and **(D)** HM-7-DNA-NC is shown; both show a balanced base composition. The *X*-axis was the base position on the reads, and the *Y*-axis was the percentage of the corresponding base at each position. A, C, G, T, and N represent different bases.

Supplementary Table 1 The peak information of HM-7-DNA.

Supplementary Table 2 The peak information of HM-7-DNA-NC.
