# Corrigendum: Purification and Identification of miRNA Target Sites in Genome Using DNA Affinity Precipitation

**DOI:** 10.3389/fgene.2020.00909

**Published:** 2020-08-21

**Authors:** Yu Xun, Yingxin Tang, Linmin Hu, Hui Xiao, Shengwen Long, Mengting Gong, Chenxi Wei, Ke Wei, Shuanglin Xiang

**Affiliations:** ^1^Key Laboratory of Protein Chemistry and Developmental Biology of Education Ministry of China, College of Life Science, Hunan Normal University, Changsha, China; ^2^Medical School, Hunan University of Chinese Medicine, Changsha, China

**Keywords:** miRNA, target sites, genome, DNA, affinity precipitation

In the original article, the author names were incorrectly spelled as Linming Hu and Yinxing Tang. The correct spellings are Linmin Hu and Yingxin Tang.

The authors apologize for this error and state that this does not change the scientific conclusions of the article in any way. The original article has been updated.

