# Supplementary figures and images for: Purification and Identification of miRNA Target Sites in Genome Using DNA Affinity Precipitation
Source: Front Genet. 2019 Sep 12;10:778. doi: 10.3389/fgene.2019.00778 (PMC6751328; doi:10.3389/fgene.2019.00778)

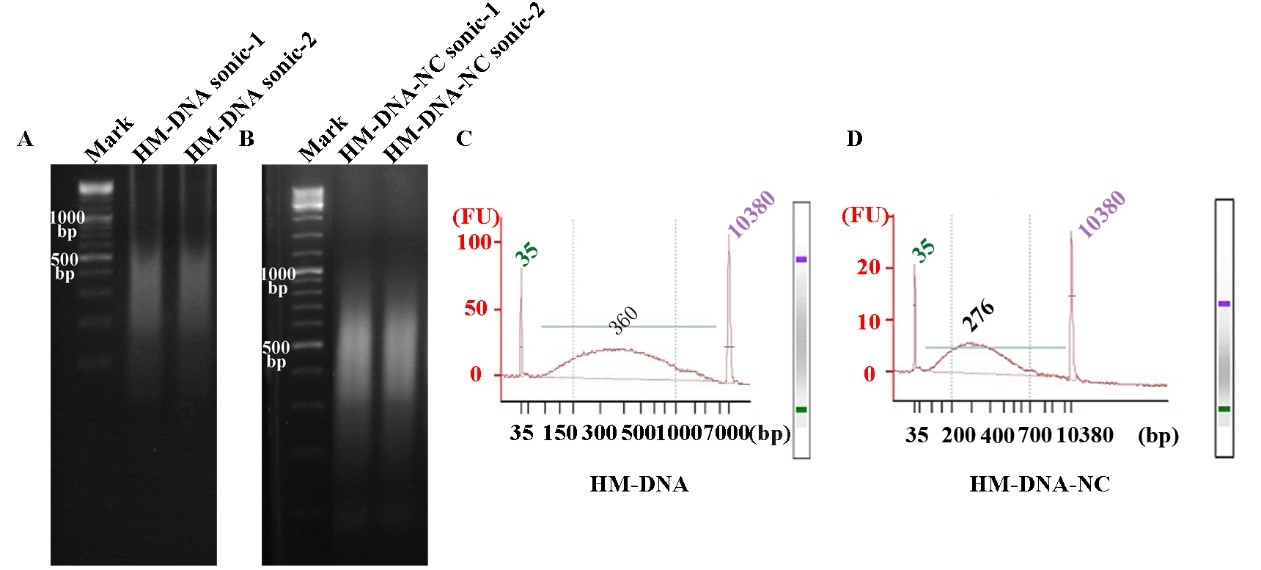

Supplement: Supplementary Figure 1 — Distribution of the main peak of genomic DNA. The genomic DNA was sheared by sonicator equipment. Then 1.5% agarose gel was used to measure the quality of samples. Results from (A) HM-DNA and (B) HM-DNA-NC are; both the main peaks of genomic DNAs were distributed between 100 and 500 bp. Agilent 2100 was used to detect specific distribution of sample fragments. HM-DNA fragment distributed at 360 bp (C); HM-DNA-NC fragment distributed at 276 bp (D). [file Image_1.jpeg]

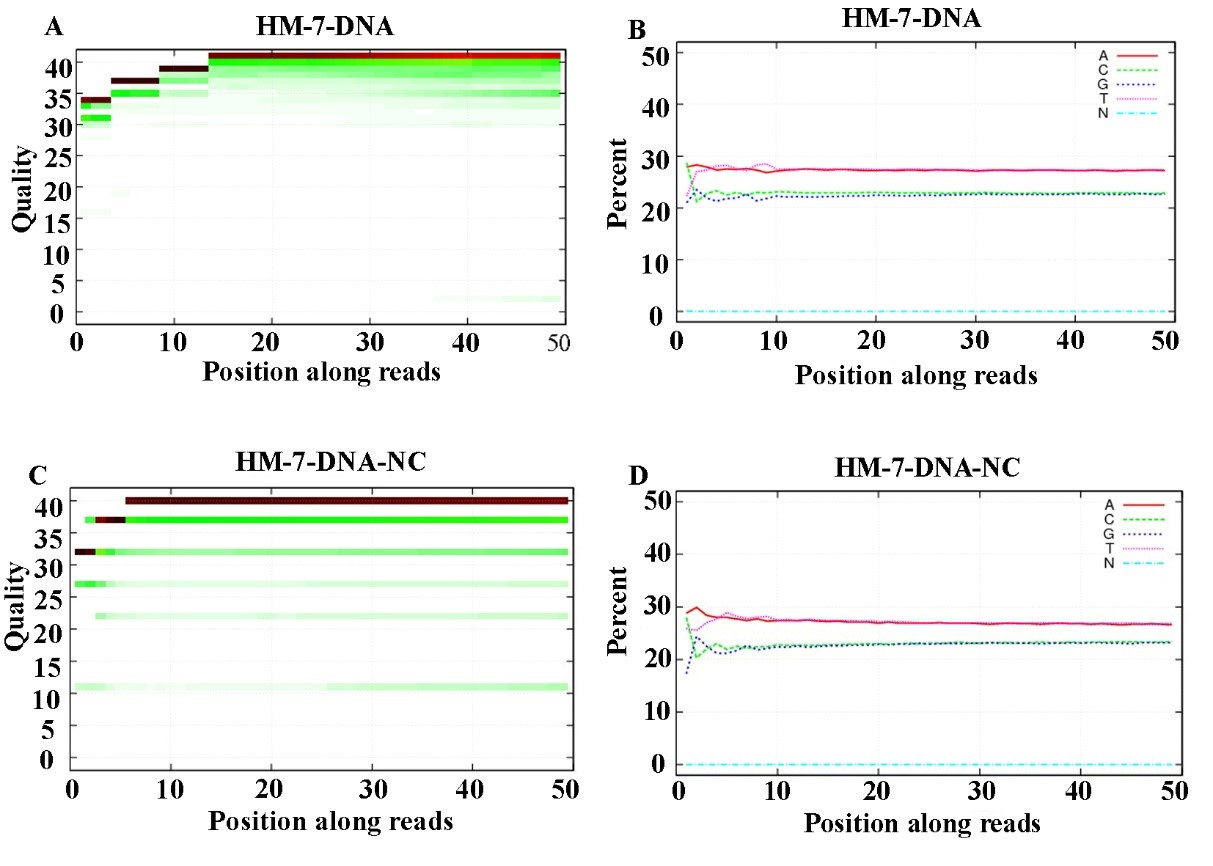

Supplement: Supplementary Figure 2 — Quality distribution and base distribution of HM-7-DNA and HM-DNA-NC. Quality distribution of (A) HM-7-DNA and (C) HM-7-DNA-NC are shown; the X-axis corresponds to the base site of the read. The Y-axis is quality value. Each dot in the image represents the quality value of the corresponding position along reads. Base distribution of (B) HM-7-DNA and (D) HM-7-DNA-NC is shown; both show a balanced base composition. The X-axis was the base position on the reads, and the Y-axis was the percentage of the corresponding base at each position. A, C, G, T, and N represent different bases. [file Image_2.jpeg]
